# nLossFinder—A Graphical User Interface Program for the Nontargeted Detection of DNA Adducts

**DOI:** 10.3390/toxics9040078

**Published:** 2021-04-07

**Authors:** Pedro F. M. Sousa, Giulia Martella, K. Magnus Åberg, Bahare Esfahani, Hitesh V. Motwani

**Affiliations:** 1 Department of Materials and Environmental Chemistry, Stockholm University, SE-106 91 Stockholm, Sweden; pedro.sousa@mmk.su.se (P.F.M.S.); magnus.aberg@mmk.su.se (K.M.Å.); 2 Department of Environmental Science, Stockholm University, SE-106 91 Stockholm, Sweden; giulia.martella@aces.su.se (G.M.); bahare.esfahani94@gmail.com (B.E.)

**Keywords:** DNA adducts, high-resolution mass spectrometry, data-independent acquisition, MATLAB, environmental monitoring

## Abstract

DNA adductomics is a relatively new omics approach aiming to measure known and unknown DNA modifications, called DNA adducts. Liquid chromatography–tandem mass spectrometry (LC-MS/MS) has become the most common method for analyzing DNA adducts. Recent advances in the field of mass spectrometry have allowed the possibility to perform a comprehensive analysis of adducts, for instance, by using a nontargeted data-independent acquisition method, with multiple precursor *m*/*z* windows as an inclusion list. However, the generated data are large and complex, and there is a need to develop algorithms to simplify and automate the time-consuming manual analysis that has hitherto been used. Here, a graphical user interface (GUI) program was developed, with the purpose of tracking a characteristic neutral loss reaction from tandem mass spectrometry of the nucleoside adducts. This program, called nLossFinder, was developed in the MATLAB platform, available as open-source code. Calf thymus DNA was used as a model for method optimization, and the overall adductomics approach was applied to DNA from amphipods (Monoporeia affinis) collected within the Swedish National Marine Monitoring Program. In the amphipod DNA, over 150 putative adducts were found in comparison to 18 using a manual approach in a previous study. The developed program can improve the processing time for large MS data, as it processes each sample in a few seconds, and hence can be applicable for high-throughput screening of adducts.

## 1. Introduction

DNA adducts are modifications on the genome resulting from the covalent binding of structural moieties on the nucleosides, or the molecular rearrangement of the nucleosides, associated with exposure to xenobiotics and endogenous processes. Liquid chromatography coupled to tandem mass spectrometry (LC-MS/MS) has become a mainstay for DNA adducts analyses [1,2,3]. An advantage of such instrumentation resides in its selectivity; for instance, high-resolution mass spectrometry (HRMS), which allows the identification of analytes and provides a straightforward quantification, as opposed to other indirect methods, e.g., ^32^P-postlabeling. The comprehensive and global analysis of DNA adducts is termed adductomics, wherein various types of DNA modifications are identified in a sample. HRMS has contributed to advances in this approach, providing accurate masses of the adduct analytes, which facilitates the identification and eventually aids in the elucidation of chemical structures, which can be employed to study trace exposure to selective chemicals [4,5,6].

Nontargeted HRMS acquisition methods applied in metabolomics or proteomics studies are being adapted for adductomics approaches, e.g., data-independent acquisition (DIA) and data-dependent acquisition (DDA). DIA is a tandem MS data acquisition method that consists of a sequential fragmentation of precursor ions, selected by specific *m*/*z* values or windows, spanning across an *m*/*z* spectrum range [7,8]. In a DDA method, the most intense scanned precursor ions are sequentially selected and fragmented [9]. The DIA method has been considered advantageous due to high accuracy and sensitivity for low-abundant analytes in metabolomics applications [7] and detecting DNA adducts [10,11]. These LC-HRMS acquisition methods tend to produce large amounts of data, and analyzing such data by a manual process, i.e., directly from the instrument software, is tedious and time-consuming. The lack of matching data from mass libraries and the unavailability of standards also pose difficulties in the identification of adducts. Several data processing methods have been developed for metabolomics and proteomics projects, e.g., Sequential Window Acquisition of all Theoretical Mass Spectra (SWATH) [12]. However, there is still a lack of available automated data analysis methods that can detect compounds through tandem reactions, such as by tracking a neutral loss under MS-CID (collision-induced dissociation), in untargeted approaches.

Our team is developing a DNA adductomics approach for ecotoxicological applications. Over the past decades, human activities have aggravated environmental pressure, including pollution load, in aquatic ecosystems worldwide, including the Baltic Sea [13]. Various chemical pollutants accumulate in sediments [14], leading to the chronic exposure of benthic fauna to sediment-bound contaminants. The Baltic amphipod *Monoporeia affinis* is a well-established sentinel species in various monitoring programs. In our previous study [6], an HRMS-based adductomics approach was employed using *M. affinis* sampled within the scope of the Swedish National Marine Monitoring Program (SNMMP), conducted by the Swedish Environmental Protection Agency. In this sample set, only 18 adducts were found by processing the DIA data manually.

In the present study, “nLossFinder,” a graphical user interface (GUI) program running under MATLAB (MathWorks Inc., Natick, MA), was developed for DNA adductomics. For the development and testing of this program, we used commercial calf thymus DNA (ctDNA) and DNA extracted from the field-collected *M. affinis* in our previous study [6]. The MS-raw data were obtained by LC-HRMS/MS, employing a DIA with sequential precursor windows method. The developed program is based on finding a neutral loss difference between precursors (MS1) and adducted nucleobase fragments (MS2) with high mass accuracy. The workflow of this approach for DNA adductomics is described along with the existing challenges. Such a software-aided tool for nontargeted DNA adducts detection is significant in the high-throughput screening of adducts in biological specimens, such as those collected in laboratory experiments and environmental surveys, and is useful in further advancing the field of DNA adductomics.

## 2. Methods

### 2.1. nLossFinder GUI and Algorithms

In untargeted analysis studies by means of LC-MS, e.g., in metabolomics or proteomics, prior to any statistical data analysis, the extraction of features from the experimental data is necessary, i.e., the identification of compounds, with attributed *m*/*z* values, distinguishable from noise. This process is not trivial due to the complexity inherent to LC-MS data, where peak shapes and background effects vary from compound to compound and from sample to sample [15]. Several feature extraction algorithms have been developed for this purpose and embedded into several open-source programs, such as mzMine [16], OpenMS [17], XCMS [18,19], or TracMass2 [20,21]. nLossFinder was developed to find neutral losses in DIA data by an untargeted approach; thus, a feature extraction method was introduced in the program to reduce the complexity of the data. This feature extraction method was adapted from TracMass2 [20] and consists of a two-step algorithm process. First, an algorithm extracts pure-ion chromatograms (PICs) from the raw data, i.e., spectral data with the same *m*/*z* values (under a tolerance) in consecutive scans throughout the chromatographic data. Then, a zero-area filter (ZAF) algorithm detects chromatographic peaks in the extracted PICs [20,22], i.e., distinguishing pure ion chromatographic peak signal data from noise. Other structural algorithms have been developed for this program, such as peak integration and other necessary GUI input/output functions. Conceptually, the data analysis strategy adapted in nLossFinder consists essentially of the following steps:

1. Separation of the raw data into one MS1 dataset and *n*-MS2 datasets (*n* is the number of DIA windows).
2. Extraction of PICs from each dataset.
3. Detection of peaks in the PICs.
4. Matching precursor peaks in MS1 with specific (adducted nucleobase) fragments peaks in MS2 that correspond to the neutral loss of interest.

nLossFinder was developed and tested in MATLAB R2020b [23], and it requires a MATLAB environment to run. The source code is provided under General Public License (GNU) version 3. A graphical user interface toolbox, GUI Layout Toolbox [24], is also required to be installed in MATLAB to run nLossFinder. The program was developed to analyze LC-MS/MS with DIA method data acquired using a Thermo Fisher (Waltham, MA) Orbitrap Q Exactive HF mass spectrometer. The experimental data must be converted from the instrument format (Thermo Fisher RAW) into mzXML format. This can be accomplished using a data format conversion program such as ProteoWizard MSConvert [25,26]. Detailed instructions on the prerequisites, installation and operation of the program are presented in the Supplementary Information.

### 2.2. Experimental

Chemicals and other materials. 2′-Deoxyguanosine (dG), 2′-deoxycytidine (dC), 2′-deoxyadenosine (dA), thymidine (dT), 5-methyl-2′-deoxycytidine (5-me-dC), *N*^6^-methyl-2′-deoxyadenosine (*N*^6^-me-dA), 8-oxo-7,8-dihydro-2′-deoxyguanosine (8-oxo-dG), deoxyribonucleic acid from calf thymus (ctDNA) sodium salt, nuclease P_1_ from *Penicillium citrinum* (NP1), phosphodiesterase I from *Crotalus adamanteus* (snake) venom (SVPDE), alkaline phosphatase from *Escherichia coli* (AKP), ammonium acetate, ammonium bicarbonate, tris(hydroxymethyl)aminomethane (Tris-buffer, pH 7.4), zinc chloride, and formic acid were obtained from Sigma-Aldrich (St. Louis, MO, USA). All solvents used were of HPLC grade. Experiments concerning DNA were carried out in DNA LoBind tubes (1.5 mL) (Eppendorf).

Sample preparation. Two kinds of samples were employed in the development and testing of the program as follows. (i) ctDNA (15 µg) dissolved in Tris-buffer (1 mM, pH 7.4, 300 µL) was enzymatically digested as described earlier [6]. (ii) A pool of digested DNA obtained in our earlier study [6] from the Baltic amphipod *M. affinis* (12 healthy females with low embryo aberrations; ≤5%) collected along the Swedish coast from the Quark in the north of the Bothnian Sea to Western Gotland Basin as a part of the SNMMP. DNA purity was determined with a Nanophotometer^TM^ (Implen, Westlake Village, CA), where the ratio A260/A280 was 1.7‒1.8 in all samples [6].

Liquid chromatography conditions. The LC-MS/MS system is composed of a Dionex UltiMate 3000 LC device coupled with an Orbitrap HRMS (Thermo Fisher Scientific, MA). The mobile phase of the LC system was a mixture of water and methanol: phase A with 5% methanol and phase B with 95% methanol, modified with 0.1% formic acid. The HPLC column used was a Supelco (Bellefonte, PA) Ascentis Express F5 (2.7 µm, 15 cm × 2.1 mm) obtained from Sigma-Aldrich. The column temperature was maintained at 25 °C and a flow rate set to 125 μL/min. The sample injection volume was 10 μL, which corresponded to 3.3% of the processed sample (300 µL). The chromatographic separation was performed with a gradient starting at 5% of B until 2 min; 2‒9 min, increasing to 30% B; 9‒15 min, increasing to 100% B; 15‒20 min, maintaining 100% B; 20‒21 min, decreasing to 5% B; 21‒25 min, maintaining 5% B.

HRMS analysis. HRMS analyses were performed using an Orbitrap Q Exactive HF mass spectrometer with a heated electrospray ionization (HESI) source in positive mode. MS parameters: capillary temperature, 275 °C; auxiliary gas, 10 arbitrary units; probe heater temperature, 240 °C; spray current, 22 µA; spray voltage, 3.5 kV; and S-Lens RF level, 60%. The tandem method (DIA) was set to include full MS1 acquisition scans, and the CID voltage was set to 30 eV. The MS1 resolution was set to 1.2 × 10^5^; automatic gain control (AGC) target, 3 × 10^6^; scan range from *m*/*z* 110 to 1200; and maximum ion injection time (IT), 200 ms. The MS2 resolution was set to 60,000 and the AGC target to 5 × 10^5^. Different DIA window widths and numbers of windows were set in different analyses. The ctDNA sample was analyzed in six experiments, using different DIA window settings with widths of *m*/*z* 5, 10, 20, 50, 100, and 350. The amphipod DNA sample was analyzed with a DIA window wideness of *m*/*z* 20, ranging from *m*/*z* 190 to 370.

## 3. Results and Discussion

**Figure 1:**
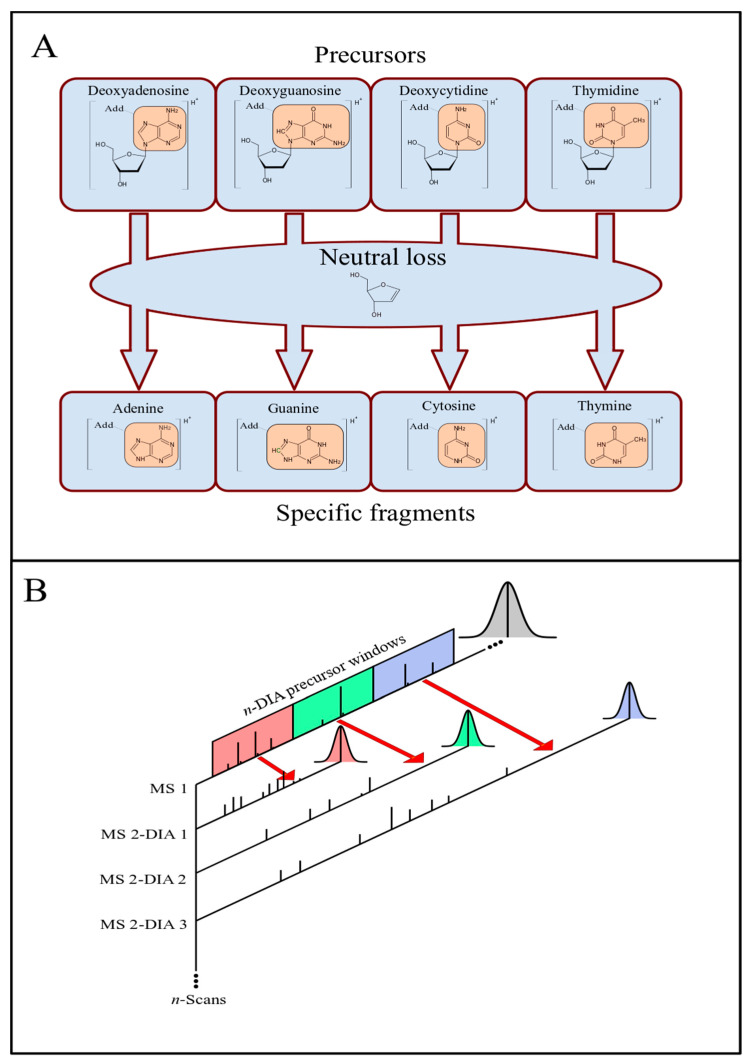
Analysis of DNA adducts by LC-MS/MS with the data-independent acquisition (DIA) method. (**A**) Fragmentation: nucleoside adducts (modification represented as “Add”) and respective CID tandem MS products as nucleobase adducts, with a deoxyribose neutral loss. (**B**) DIA method: precursor ion scans (MS1) are followed by sequential CID product ions scans within specific precursor *m*/*z* windows. The scan sequence is repeated throughout the LC elution process.

The fragmentation of ionized adducts on the nucleosides (dA, dG, dC, and dT) by tandem mass spectrometry (MS/MS) can occur with the neutral loss of deoxyribose, resulting in the corresponding ionized bases adenine (A), guanine (G), cytosine (C), and thymine (T) with the specific adduct moiety (Figure 1A). Thus, a potential DNA adduct is considered detected when the difference between a precursor and a specific fragment corresponds to the deoxyribose neutral loss (116.0473 Da). Based on this fragmentation, the approach to employing a DIA method with sequential precursor dissociation spanning over *m*/*z* windows is illustrated in Figure 1B.

For each DIA window, a set of precursor ions, within a determined *m*/*z* range, is selected and fragmented. The number and the width of the precursor windows span the *m*/*z* range of the precursor ions. Throughout a chromatographic run, *m*/*z* spectra scans are recorded in sequences of blocks. In each block, the first scan corresponds to a full-MS1 precursor ion spectrum, and the following scans are full-MS2 spectra from the fragmentation products of precursors within each DIA *m*/*z* window range, i.e., each MS1 is followed by *n*-MS2 scans, where *n* corresponds to the number of precursor DIA windows (Figure 1). The data obtained by this method can be analyzed manually using the instrument software (e.g., Thermo Fisher Xcalibur Qual Browser). However, this process can be more efficient and less time-consuming if performed computationally. Thus, a user-friendly GUI program with embedded algorithms, called nLossFinder, was developed for such purpose in this work.

The peak detection by nLossFinder is performed in two steps, which depend on parameters defined by the user in the GUI. As mentioned under the Methods section (Section 2), first, PICs are extracted from the raw precursor (MS1) and tandem (MS2 for each DIA window) data. Then, a matching filter algorithm is employed to detect peaks in the PICs. A peak is detected when a part of the signal data in each PIC has a Gaussian shape, or alike, i.e., parts of the signal that have an increasing slope, followed by a decreasing slope, that can be distinguished from noise. The number of data points that constitute a peak should be higher than five for optimal identification, although less resolved peaks (three points) can also be detected, depending on the GUI settings. Other parameters can be set to modulate the sensitivity of the matching filter, such as the signal-to-noise ratio, which can determine the sensitivity of the peak detection relatively to chemical noise.

During the development of nLossFinder, initially, ctDNA was used as a mechanistic model sample. The nucleoside mixture obtained by the digestion of ctDNA was analyzed by LC-HRMS/MS using the DIA method, varying the number of precursor windows and the window width. These experiments, summarized in Table 1, were performed to assess the quality of the results while varying the number of DIA windows (*n*, cf. Figure 1) and the window widths. The results demonstrate that too many DIA windows, with widths as narrow as *m*/*z* 5, or too few DIA windows, as wide as *m*/*z* 100 or 350, result in a lower number of potential matches. The quality of the results was assessed based on total putative adducts found and the peak shapes observed in nLossFinder. Good peaks (in Table 1) are peaks that have a regular Gaussian shape and are not noisy (with spikes). With wider DIA windows, the peaks become increasingly noisy.

In our previous study [6], the number of DIA windows was in the form of a CT10 experiment (Table 1), but the interpretation of the results was performed manually. An approach such as in CT5 and CT10, i.e., many and narrow DIA windows, is, in principle, helpful to identify DNA adducts manually with the instrument software (Xcalibur Qual Browser). When using an algorithm, such as in nLossFinder, where visualization is not a concern for finding neutral losses, few and wider windows increase the number of spectral points per peak. The identification of peaks of abundant compounds may not be much affected by a low scanning frequency, for instance, as that with 30 DIA windows set in the analysis, spectral data are recorded once every 30 scans in the precursor (MS1) and in each fragment (MS2) chromatograms (cf. Figure 1). However, less abundant compounds may be characterized by too few spectral signals, which may pose difficulties in the identification of features (peaks). Further, very-wide windows (>*m*/*z* 50) resulted in noisy peaks, and the number of adducts detected with nLossFinder was low compared to the narrower windows. These results suggest that there are limits at an instrumental level for this approach, where very-wide DIA windows may compromise the quality of the results. Thus, according to these experiments, more putative adducts with good-quality peak shapes were detected with nLossFinder when the precursor window width was set to *m*/*z* 10 or 20, and a number of DIA windows (*n*) of 16 or 9, respectively, and a precursor center from *m*/*z* 200 to 350.

**Figure 2:**
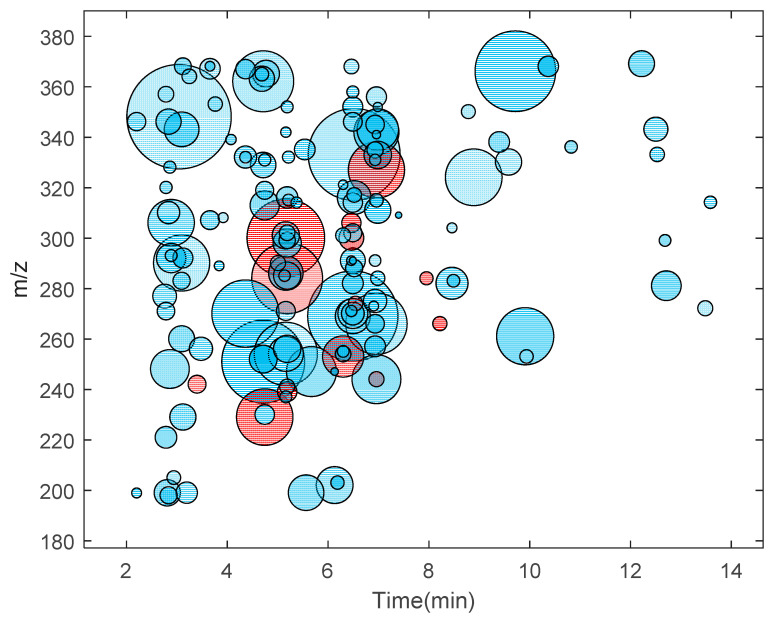
An adductome map (*m*/*z* of detected adduct ions vs. elution time) of the putative DNA adducts found in *M. affinis* (pool of 12 individuals) using nLossFinder shown as blue circles. The areas of the circles are proportional to the area of the adduct ion peaks (ranging from 1.8 × 10^4^ to 1.2 × 10^8^ peak area units). The circles in red correspond to the adducts that were found manually in a previous study (Gorokhova et al. [6]). For a list of putative adducts in *M. affinis* detected in this study and earlier [6], refer to the Supplementary Information (Table S1).

The DNA adductomics approach was applied to *M. affinis*, using the conditions optimized with the ctDNA digest sample. The DNA from the sampled amphipods was extracted and digested in our earlier study [6]. A pool of digested DNA from 12 individual amphipods was analyzed by using the DIA settings which gave the most optimal results according to Table 1, i.e., nine windows, width *m*/*z* 20, and precursor center from *m*/*z* 200 to 350. This data set was analyzed in nLossFinder, and a putative list of 153 DNA adducts was generated. Possible ESI adducts and isotopes were not removed from the list though. A table with the list of putative adducts found in *M. affinis* is presented in the Supplementary Information (Table S1). These adducts found in *M. affinis* are represented in the form of an adductome map in Figure 2. The map is created by plotting all detected adducts, *m*/*z* values of molecular ions against their corresponding elution time (retention time on the chromatogram). This type of adductome mapping can be useful as the fingerprint of all background DNA modifications in the specific species from a particular location and provide information for establishing the background assessment criteria [27] based on the detected adducts.

The adducts found in *M. affinis* samples included the epigenetic markers 5-me-dC and *N*^6^-me-dA and the oxidative stress marker 8-oxo-dG, which were confirmed by comparison with reference compounds as shown in our previous study [6]. In addition, four other modifications (5-OH-dC, dU, dI, Gh, cf. Table S1) tentatively identified earlier [6], were found in the present study. In an earlier study on the same type of sample, which was performed in 2018 [6], when manual processing was employed, in total 18 putative adducts were detected, of which 6 (Table S2) were not found using nLossFinder in the sample analyzed in this work. This was confirmed manually using the software from the instrument (Thermo Xcalibur—Qual Browser), which indicated that these adducts may be unstable when stored in the resulting matrix at −20 °C for nearly two years.

**Figure 3:**
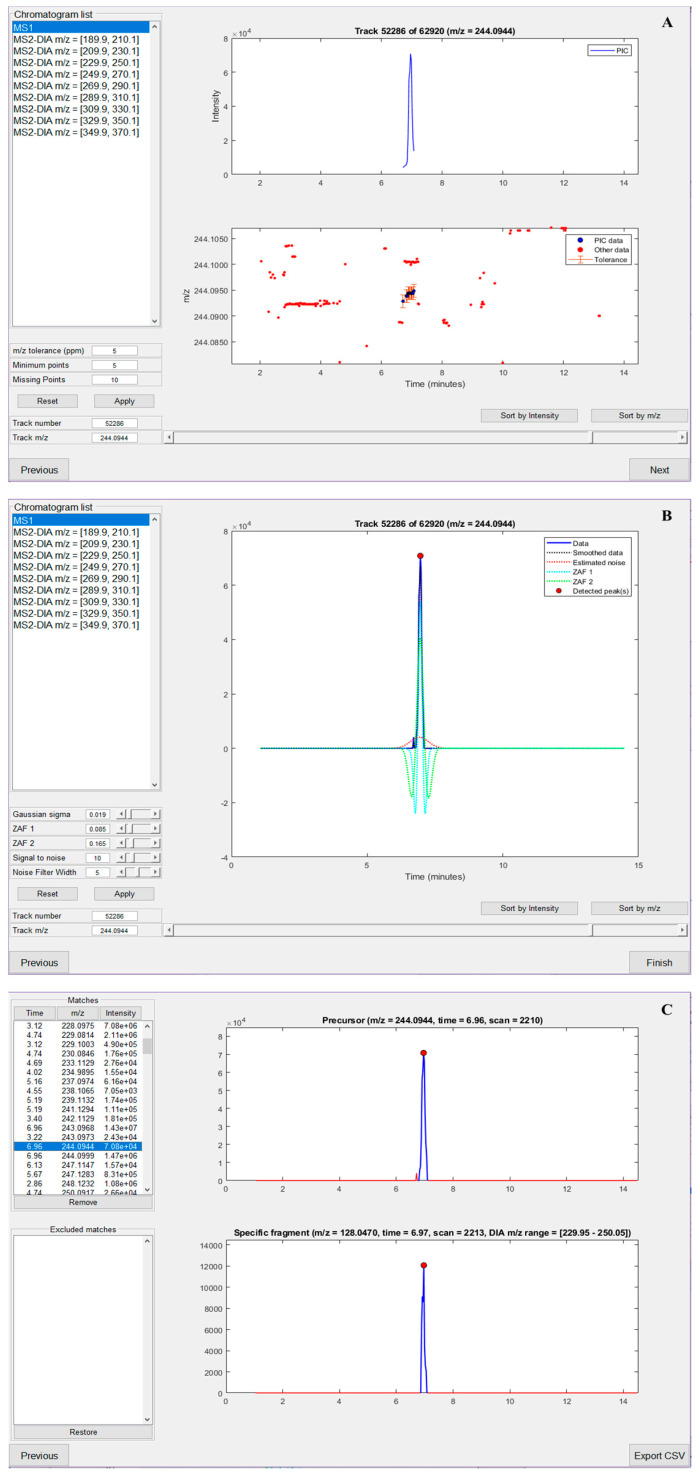
nLossFinder GUI sections (illustrated for analyzing the adduct 5-OH-dC). (**A**) Pure ion chromatogram (PIC) extraction parameters setup. (**B**) Zero-area filter (ZAF) peak detection parameters setup. (**C**) Analysis of processed results.

To summarize key features of nLossFinder, prior to the data processing, i.e., finding adducts, the user must set up peak detection parameters. New parameters should be set for different experimental conditions (e.g., different number of DIA windows and widths, and chromatographic elution time window). The parameters for the extraction of PICs (Figure 3A, exemplified for 5-OH-dC) define the minimum number of points in PIC, tolerance for missing data points within a PIC, and *m*/*z* error tolerance. The parameters for peak detection define how the ZAF will detect peaks in the extracted PICs (Figure 3B). There is a detailed explanation of the steps and the parameters used in the peak detection algorithms of nLossFinder in the Supplementary Information. After processing the experimental data, the user can visualize the detected adducts as a list, with the options to sort the results by retention time, *m/z*, or intensity values of the precursors (Figure 3C). It is possible to visualize and discard unwanted matches, such as potential ESI adducts, isotopes, or matches that may seem not desirable, e.g., very low intensity, with irregular peak shapes, or somewhat noisy, etc. Furthermore, the analysis of the results can be performed elsewhere, i.e., a list containing retention time, *m*/*z*, intensity, and peak area values of the precursors and specific fragments, as well as the number of data points per peak, is exported as a comma-separated values (CSV) file, which can be opened in another program (e.g., Microsoft Excel) for further analysis. nLossFinder is rapid in processing the data and thus can provide high-throughput results, although each sample must be analyzed separately. The processing time of the *M. affinis* sample data was 36 s in a PC (Shark Gaming Systems, Glostrup, Denmark) equipped with Intel(R) Core(TM) i9-9900K CPU @ 3.60 GHz, 16 GB RAM, and a 4 GB GPU.

A common disadvantage of using computational programs for the screening of certain chemicals/adducts is that they may generate false positives. These may arise, for instance, if there are artifacts that can mimic the deoxyribose neutral loss, e.g., neutral losses of isomers of deoxyribose, or neutral loss from multiple charged precursors. Because reference compounds are seldom available, the user must set some criteria in the interpretation of the results. Some criteria that the user can adopt to minimize the number of false-positive matches while using nLossFinder are to verify the peak shapes of precursor and specific fragments in each match and discard matches that the program has found but have irregular shapes or do not overlap properly. An algorithm in nLossFinder automatically performs such tasks using peak shapes as a filtering criterion to reduce false positivity. This algorithm is employed to separate the detected peak data from the rest of the PIC data (surrounding noise). The process is accomplished by applying a smoothing filter on each detected peak apex and intersecting the local minima of the smooth peak with the PIC data. This extracts the peak data that have the best shape, discarding the rest of the PIC data (noise before and after the peak). This algorithm also checks for local maxima around the peak, which can reveal that the peak is surrounded by high PIC noise signals. Moreover, the overlapping between precursors and specific fragment peaks is also verified. Matches that do not meet the criteria (shape and overlap) are discarded automatically. However, a final visual review of the matches is recommended to be performed in nLossFinder to confirm that all the matches have desired shapes, proper overlapping, etc. (such as in Figure 3C). Some matches may eventually pass these filters, such as very-low-abundant precursors that may be characterized by multiple small consequent peaks. In the total findings presented in Table S2 (about 150 putative adducts), only a few low intense matches (about 5) had to be removed manually. The visual confirmation also allows studying the matches that correspond to isotopes and ESI adducts, which are considered to be false positives. Nonetheless, this latter process can be performed elsewhere from the output table.

The isotopes can be useful to determine the precursor ion charge but cannot be taken as a positive match and, therefore, should be excluded. Moreover, ESI adducts such as sodium, ammonium, potassium, etc., if already found in their protonated form, should not be considered as additional adducts. Isotopes and ESI adducts can be identified by sorting the precursors by retention time and comparing their *m*/*z* values in overlapping matches. However, when samples in a study are prepared and processed in the same way, the false positives may not have any significant impact, e.g., in identifying adducts that are linked to a disease state or to a particular exposure. Nevertheless, if required for selective purposes, a targeted acquisition method, e.g., parallel reaction monitoring, could be applied on the generated list of putative adducts to increase the confidence in the identification of the putative adducts and to aid in ruling out the false positives from the untargeted DIA approach. In the future development of nLossFinder, it is planned that the isotopes and ESI adducts will be removed or highlighted. It is planned to be able to analyze batches of samples, rather than one at a time, as nLossFinder is processing presently. This will allow comparison with different samples, including those with background samples, when available, to identify and remove false positives.

## 4. Conclusions

A program (nLossFinder) with a GUI was developed for the detection of DNA adducts, which has a scope to advance the field of DNA adductomics. This open-source program (download link in the Supplementary Information) can aid in the discovery of DNA adducts from the analysis of DNA digests by means of LC-HRMS/MS using a DIA method. The number of DIA windows and the window width were studied by comparison of the results obtained in six experiments, using ctDNA digest, which was analyzed with different DIA parameters. With nine DIA windows and a width of *m*/*z* 20, the highest number of putative DNA adducts were discovered with relatively good peak shapes. The optimized method was applied to an environmental sample consisting of a DNA digest pool from 12 Baltic amphipods *M. affinis*, in which about 150 putative adducts were detected. These included biomarkers for epigenetic changes (5-me-dC and *N*^6^-me-dA) and oxidative stress (8-oxo-dG), which was in agreement with a previous study wherein manual processing was performed on the same type of samples of *M. affinis*, but only a few adducts (ca. eight times less) were found. These initial results suggest that DNA adductomics could be developed as a proactive tool in assessing the health of the wild population from exposure to contaminants.

## Figures and Tables

**Table 1:** Evaluation of nLossFinder from running at different DIA windows settings using a nucleoside mixture obtained from the digestion of ctDNA. ^a^ Experiments were named CT5, CT10, and so on based on the respective window width. ^b^ Only precursors above 10^4^ peak area units were considered. Possible isotopes and ESI adducts were not removed.

Experiment ^a^	Number of Precursor DIA Windows	Window Width (*m*/*z*)	Precursor Range (*m*/*z*)			Total Putative Adducts Found ^b^	Peak Quality
CT5	31	5	197.5	-	352.5	68	Good
CT10	16	10	195	-	355	115	Good
CT20	9	20	190	-	370	162	Good
CT50	4	50	175	-	375	64	Slight noisy
CT100	3	100	150	-	450	55	Moderate noisy
CT350	1	350	175	-	525	14	Very noisy

## Data Availability

The data presented in this study are available in the main article and supplementary material. Download link of the developed program is provided in the Supplementary Material.

## Supplementary Materials

The following are available online at https://www.mdpi.com/article/10.3390/toxics9040078/s1, nLossFinder User Manual containing download link to the program source code, Table S1: Output list of the putative DNA adducts found in *M. affinis* using nLossFinder, Table S2: Putative DNA adducts found in *M. affinis* using a manual approach that were not detected in the samples analyzed in this work.
