# Supplementary material for: nLossFinder—A Graphical User Interface Program for the Nontargeted Detection of DNA Adducts
Source: Toxics. 2021 Apr 7;9(4):78. doi: 10.3390/toxics9040078 (PMC8067598; doi:10.3390/toxics9040078)
Supplement: Supplementary file 1 [file toxics-09-00078-s001.pdf]

nLossFinder –

A graphical user interface program for non-targeted detection of DNA adducts

Supplementary Information

nLossFinder User Manual

Requirements and installation

The nLossFinder program requires a MATLAB environment (R2020a or later) to work, and also the installation of the MATLAB add-on ‘GUI Layout Toolbox’, which can be downloaded from the MATLAB website https://se.mathworks.com/matlabcentral/fileexchange/. nLossFinder was tested in Windows 10, running on a PC equipped with Intel(R) Core(TM) i9-9900K CPU @ 3.60GHz, 16 GB RAM and 4 GB GPU. Inferior hardware should work fine, although minimum 8 GB RAM is recommended.

Download the nLossFinder source code from https://github.com/pfsousa77/nLossFinder. To install nLossFinder, unpack (unzip) the code files into any folder accessible by MATLAB. In MATLAB open this folder and type ‘nLossFinder’ in the Command Window to run.

nLossFinder was designed to analyze LC-MS/MS data using a DIA (data independent acquisition) method and has only been tested on data obtained from a Thermo Fisher Orbitrap Q Exactive HF mass analyser. The experimental files obtained from the instrument (Thermo Fisher RAW format) must be converted into mzXML format in order to analyze them in nLossFinder. This conversion can be performed using ProteoWizard MSConvert, which is an open source program that can be downloaded from the website http://proteowizard.sourceforge.net/. The MSConvert parameters must be set as illustrated in Figure S1, where, from the generic defaults, the output format is ‘mzXML’, the ‘zlib compression’ is unchecked, and a ‘Peak Picking’ filter (MS levels 1-2) is added. This filter centroids the data.

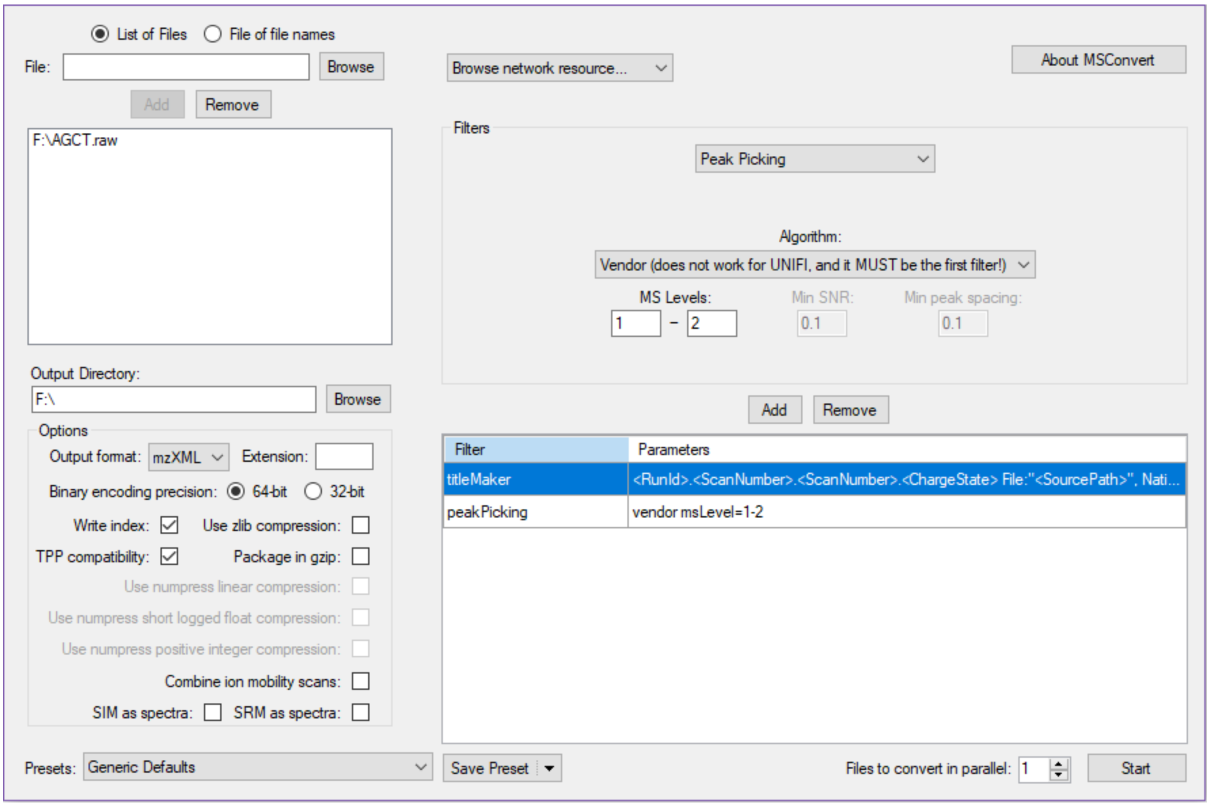

Figure S1. ProteoWizard MSConvert setup.

nLossFinder - Main menu

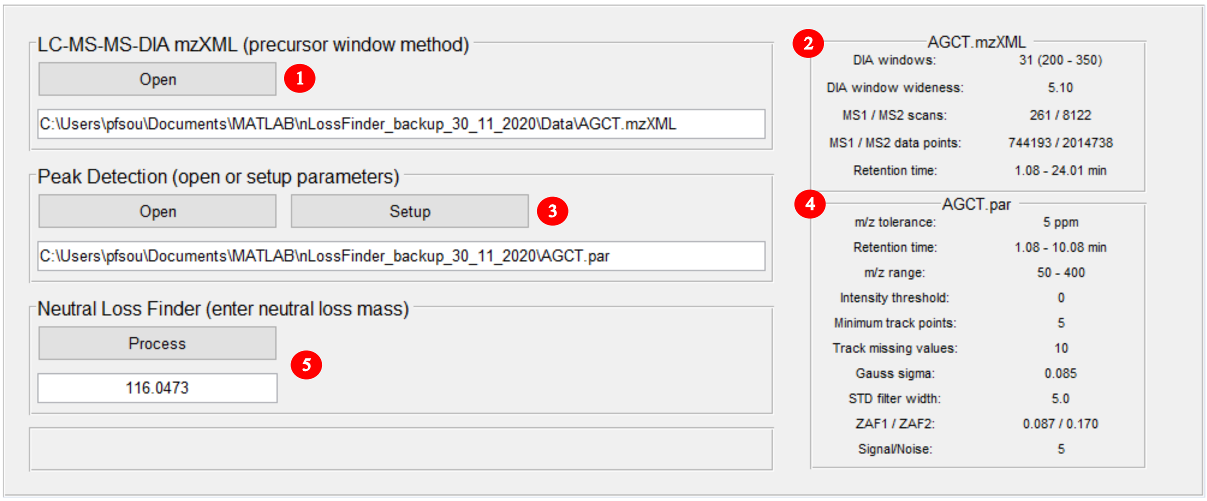

Figure S2. nLossFinder main menu.

The main menu of nLossFinder appears as shown in Figure S2. The panels 1–5 are described below.

(1) Open a mzXML file containing LC-MS/MS data obtained using the DIA method.

(2) If the file is opened successfully, the information of this file will appear in the panel.

(3) Setup or open peak detection parameters. The peak detection parameters must be setup for different experimental conditions. Otherwise, one setup file can be applied in a batch of experiments. The GUI continues to the peak detection steps explained bellow.

(4) The peak detection parameters are displayed in the panel to the right.

(5) After setting up the peak detection parameters, enter the value of the neutral loss (*i.e.* deoxyribose, 116.0473 Da), then click to process the peak detection and neutral loss finder algorithms. After processing, a new section will open to visualize and review the results (see below nLossFinder - Analysis of Results).

Peak detection setup – retention time range

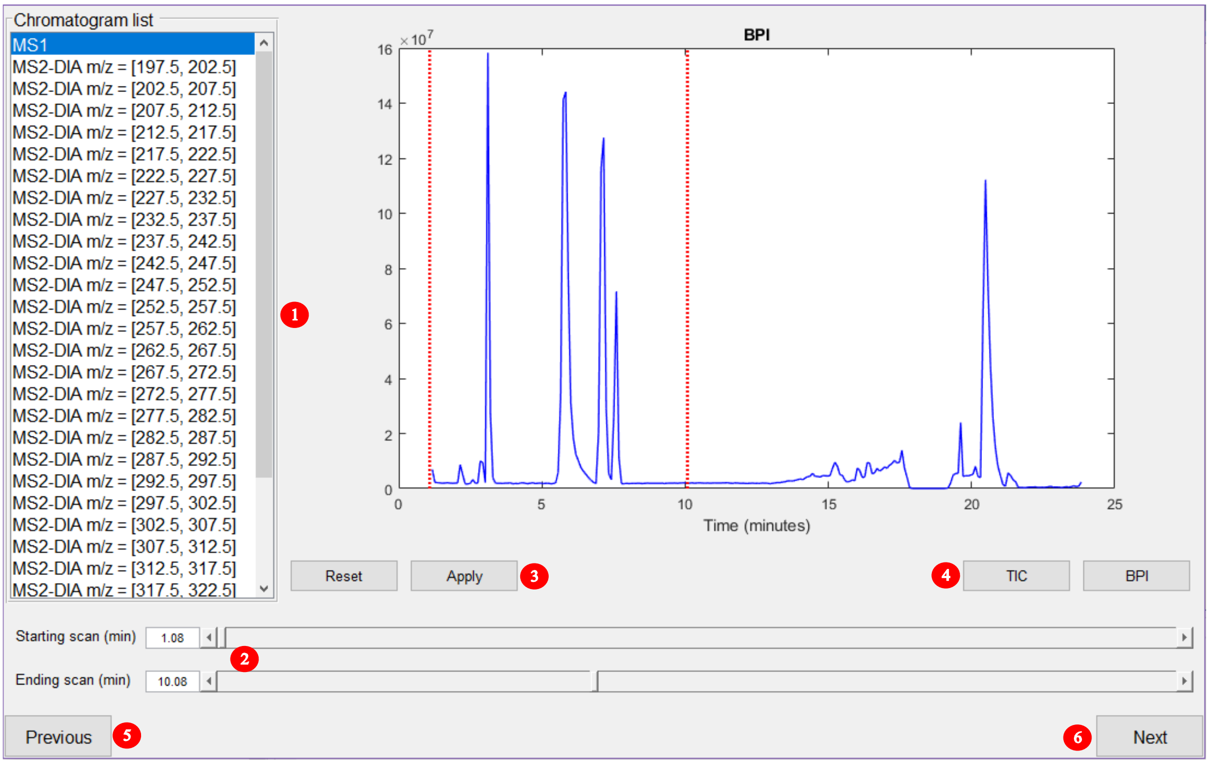

Figure S3. Setup retention time range.

(1) The user can visualize the chromatograms, *i.e.*, full scan MS1 and MS2 DIA (Figure S3).

(2) Sliding the bars, or using the arrows, or input starting and ending retention times will subset all the chromatograms.

(3) Use the buttons 'Reset' or 'Apply' on the selected scan range.

(4) Visualize Total Ion Chromatograms (TIC) or Base Peak Chromatograms (BPI).

(5) Go back to main menu.

(6) Apply settings and move to the next section.

Peak detection setup – *m/z* range and precursor minimum intensity threshold

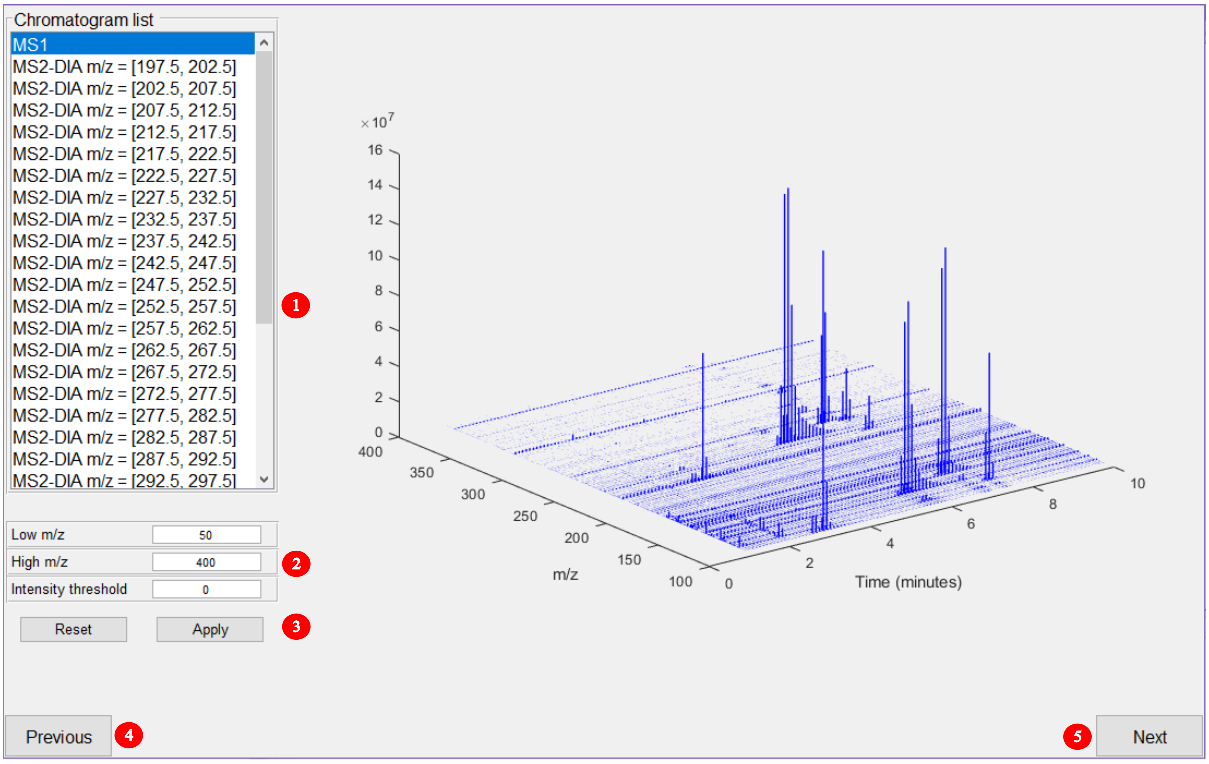

Figure S4. Setup *m/z* range and minimum intensity threshold.

(1) The user can visualize the spectral maps for each chromatogram, *i.e.*, full scan MS1 and MS2 DIA (Figure S4).

(2) Set the lowest and highest *m/z* value, and the minimum intensity. This only affects the precursor (MS1) spectra though.

(3) Undo or apply the settings.

(4) Go back to the previous section.

(5) Apply settings and move to the next section.

Peak detection setup – Pure Ion Chromatogram (PIC) tracker

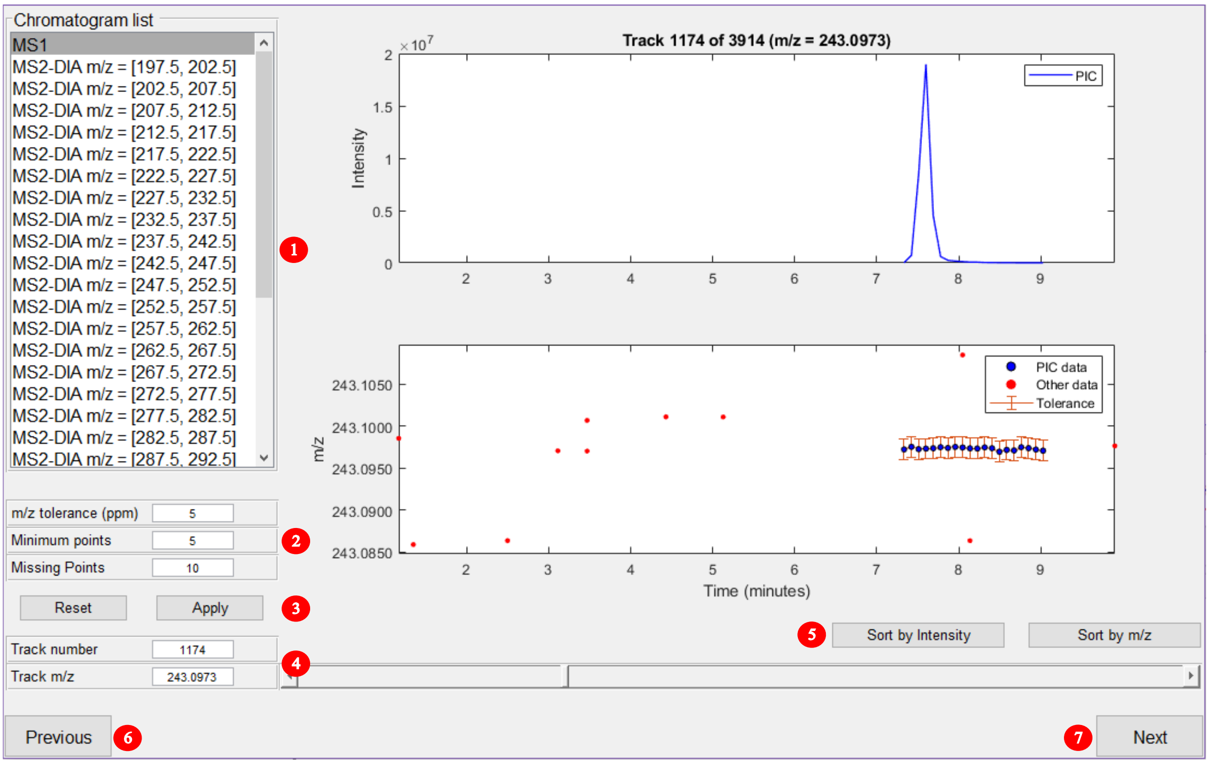

Figure S5. Setup PIC tracking parameters.

(1) Select chromatogram, *i.e.*, full scan MS1 or MS2 DIA (Figure S5).

(2) The *m/z* tolerance sets the desired tolerance for tracking *m/z* values across scans. This value is the same used when processing the data (from the main menu) to find the neutral loss matches between precursor and fragment ions. The minimum points define how many points a PIC should have, and the missing points is a tolerance for missing *m/z* values between scans.

(3) Undo or apply the settings. Here the spectral data that does not belong to any PIC are discarded.

(4) Input *m/z* values to explore the tracked PICs.

(5) Sort the tracks by intensity or by *m/z* values. This and the latter (4) are useful when searching of an ion of interest, *e.g.*, type a *m/z* value around the value of interest and move the arrows until the actual *m/z* value is found.

(6) Go back to the previous section.

(7) Save settings and move to the next section.

Peak detection setup – Peak detection

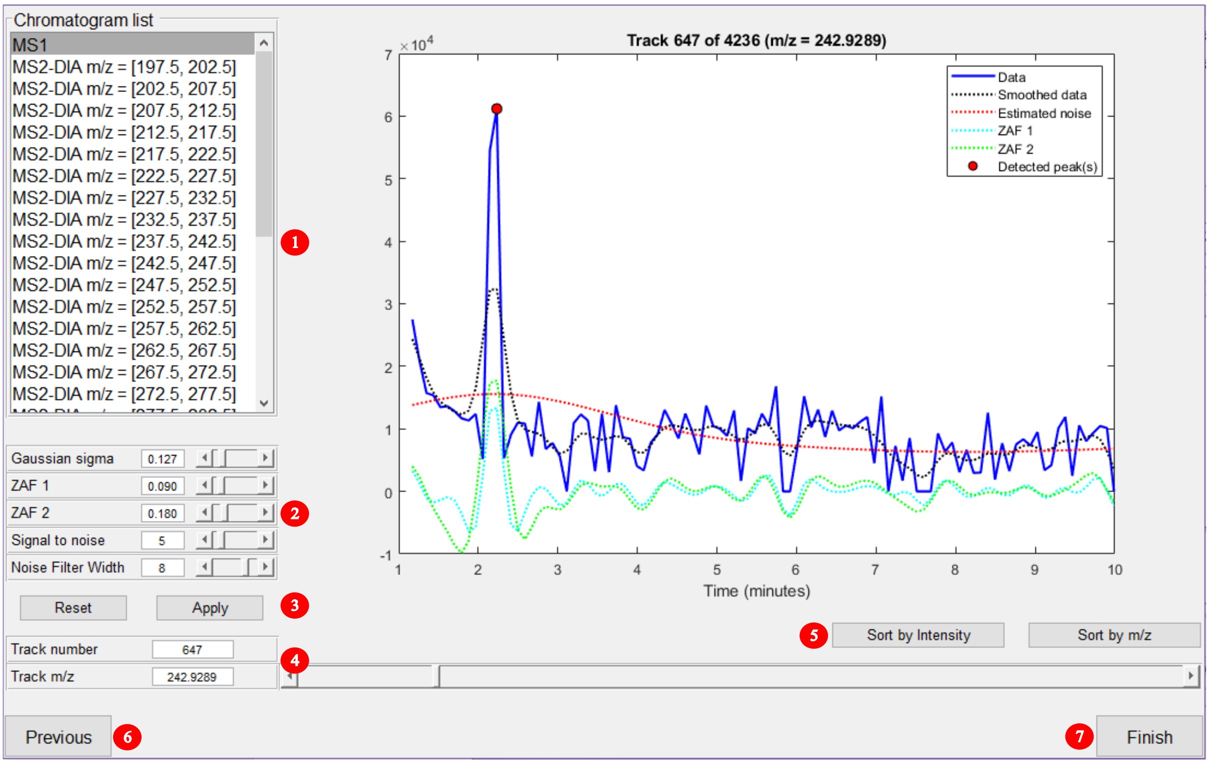

Figure S6. Setup peak detection parameters.

(1) Select chromatogram, *i.e.*, full scan MS1 or MS2 DIA (Figure S6).

(2) The control box contains parameters that determine the peak detection. A matching filter is applied on the PICs to detect peaks, if any. The criteria, of the matching filter, is the application of a fitting curve convoluted with the PIC intensity vector (over the time axis). This curve (dotted blue or green) is the second derivative of the gaussian equation, which has a sharper shape than a normal gaussian curve. Along with that, another curve is also convoluted with the data, but with the weighted standard deviation of the PIC data (red dotted line). This curve is an estimate of the noise in a PIC. The overlap between a matching filter curve(s) and the noise estimate curve determines when a peak is detected.

i. Gaussian sigma - This parameter controls the smoothed data (black dotted line) and the noise estimate (red line). By clicking on the arrows, *i.e.*, increasing or decreasing the value will modulate these lines depending on the data (blue line). For a sensitive filter, the red line should be as close to the base line as possible.

ii. ZAF1 and ZAF2 - are the Zero Areas Filters parameters that modulate the blue and green dotted lines. These filters fit the PIC data. Whenever these lines overlap the red line (estimated noise) a peak is detected, and a red circle will be plotted on the maximum of the detected peak. The ZAF 2 should be the double of the ZAF 1 value. These filters do the same, but one should be broader than the other, so that the peaks with different widths can be fitted better.

iii. The signal to noise will raise the noise estimate curve (red line) and the Noise Filter Width will broaden it. These parameters adjust the shape and height of the noise estimate curve and can be used to modulate sensitivity of the matching filters, *i.e.*, a higher estimate noise curve

will require more evidence to accept a feature as a peak, resulting in fewer and less noisy peaks. However, by doing so will also lead to a risk of missing low abundance peaks (adducts).

(3) Reset the parameters or apply. Applying will remove PICs where no peaks have been detected. This process may be slow, and it is not required to setup the parameters. In this stage, it just informs how many tracks will be used when processing to find neutral losses.

(4) Input *m/z* values to explore the tracked PICs.

(5) Sort the tracks by intensity or by *m/z* values. This and the latter (4) are useful when searching of an ion of interest, *e.g.*, type a *m/z* value around the value of interest and move the arrows until the actual *m/z* value is found.

(6) Go back to the previous section.

(7) Save the peak detection parameters and go to the main menu.

nLossFinder – Analysis of results

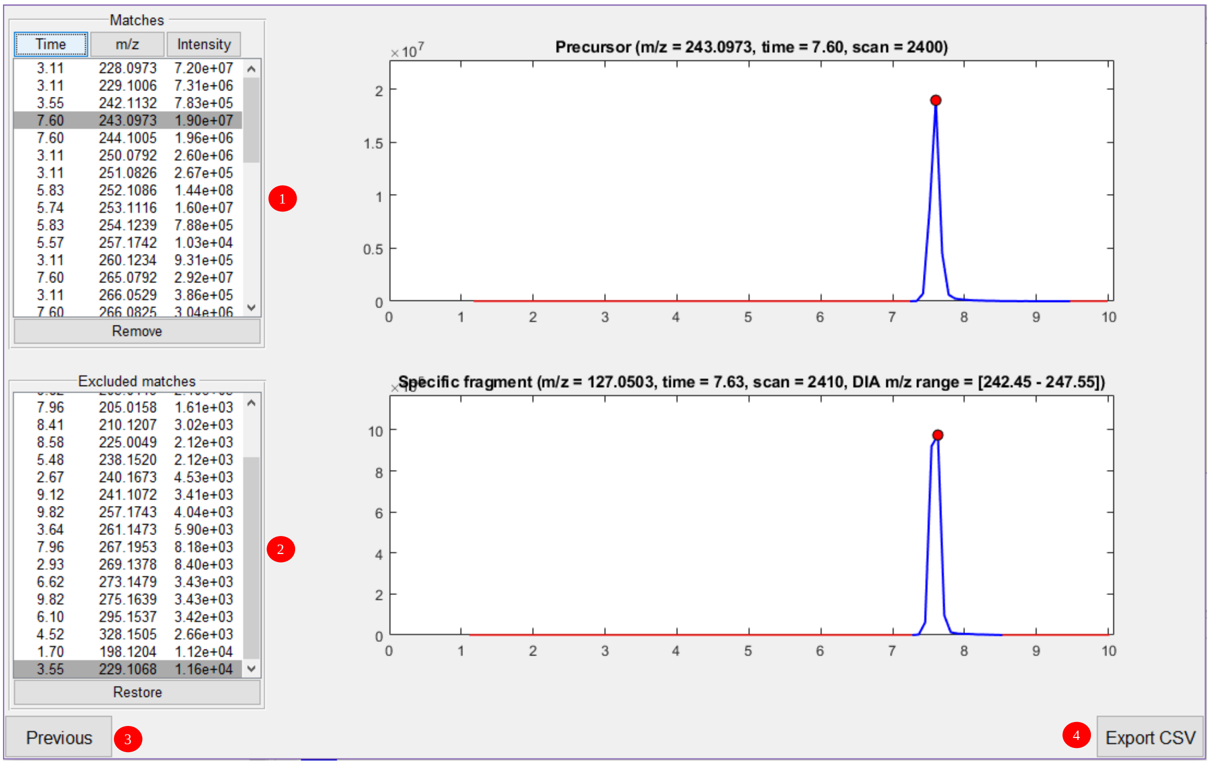

Figure S7. Analysis of matches and exporting results.

(1) The results obtained by the nLossFinder algorithms are presented in this list (Figure S7, Matches). The list can be sorted according to the retention time, the *m/z* value or the intensity at the maximum of the peak of the precursors. Sorting the precursors by intensity can help evaluate the matches of lower intensities, which are usually peaks in noisy PICs, *i.e.*, dragging signals across the chromatogram. These may be removed from the list if needed. Also, isotopes and adducts such as sodium, potassium, or other possible ions that may be formed in the ionization process can be

tracked by sorting (first my *m/z*, then by retention time). Those matches with the same (or very close) retention times are potentially isotopes and ESI adducts and can be removed if needed. This is illustrated in Figure S8. Here, 6 matches with the same retention time (7.60 min) were selected. The first match (*m/z* 243.0973) corresponds to the protonated thymidine (the sample analyzed in this experiment was a mixture of the four nucleoside standards dA, dG, dC and dT), the second (*m/z* 244.1005) is an isotope (C13) of the first ion, the third match (*m/z* 265.0792) is a sodium adduct (proton-sodium exchange, 22 Da) of the first, and the fourth (*m/z* 266.0825) is an isotope of the third. The other two may be also adducts formed in the ESI, such as dimmers or other side reactions, or eventually just overlapping compounds. Therefore, these results should be carefully evaluated, to avoid including many false positives in the results.

(2) The excluded matches list (Figure S7) will not be saved in the output data. Clicking on restore will move the match(es) back to the inclusion list.

(3) Go back to the main menu. The processed results are lost though.

(4) Save the results (Matches) in a table, in a comma separated values (CSV) file, which can be opened in Excel, or any other program for further data analysis. The output file contains the retention time, *m/z* values, intensities of the maximum of the peaks and peak areas of the precursors and specific fragments. The neutral loss value (difference between precursor and specific fragment) and the DIA value (the center of the corresponding precursor DIA window) are also recorded.

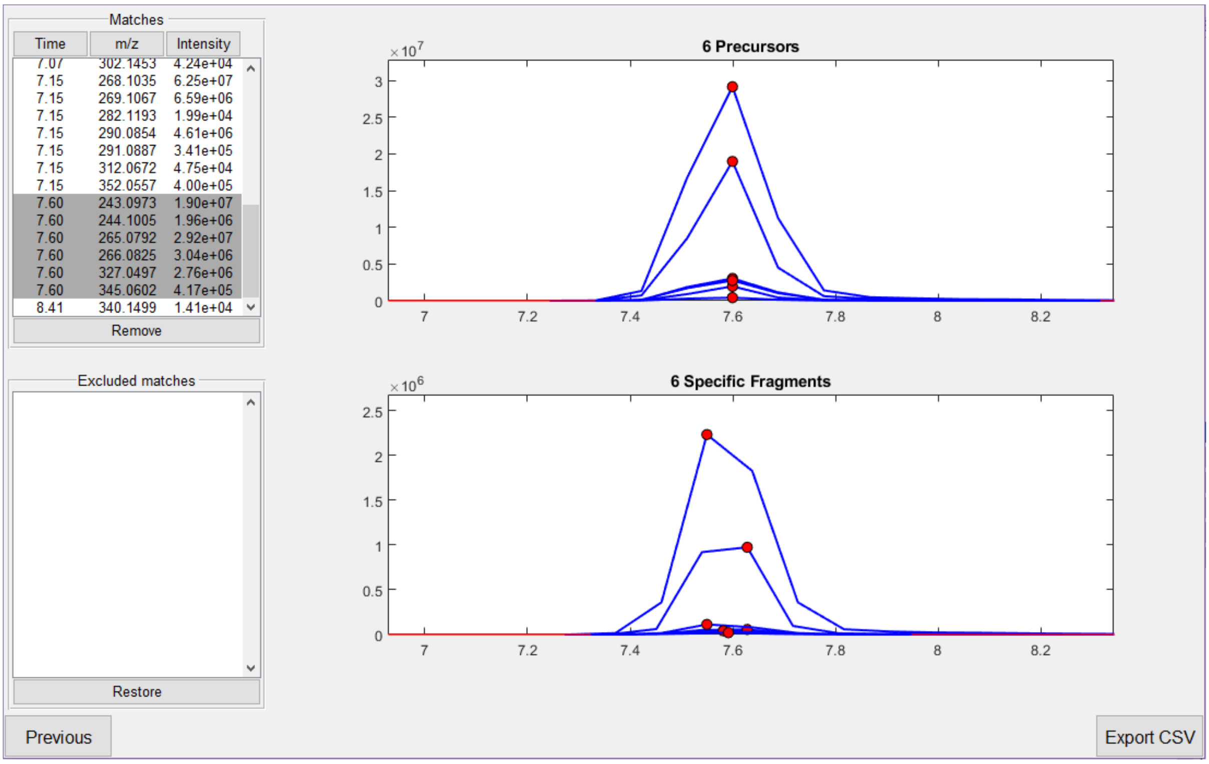

Figure S8. Potential isotopes or ESI adducts can be studied based on overlapped ion peaks.

Results

Table S1. Output list of the putative DNA adducts found in *M. affinis* using nLossFinder. Represented as adductome map in Figure 2 in the article. Precursor**[a]** and specific fragment**[b]** correspond to the nucleoside adduct ion and nucleobase adduct ion, respectively (cf. Figure 1). **[c]**The non-adducted nucleosides (dA, dG, dC, dT) and sodium adducts are not represented in the adduct map in Figure 2.

**Precursor[a] *m/z***	**Specific fragment[b] *m/z***	**Retention time (min)**	**Neutral loss (Da)**	**Neutral loss error (ppm)**	**Precursor area**	**Specific fragment area**	**Adducts detected in earlier work (Gorokhova *et al.*[16]) and their proposed identification (ID)**
198.0845	82.0375	2.83	116.0470	1.39	5.24E+05	1.83E+05	
198.9933	82.9469	2.20	116.0464	4.62	9.03E+04	1.82E+04	
199.0684	83.0214	2.81	116.0471	1.25	1.71E+06	1.09E+06	
199.1073	83.0602	3.19	116.0472	0.72	9.82E+05	3.83E+05	
199.1074	83.0602	5.56	116.0472	0.35	3.60E+06	1.23E+05	
202.1071	86.0599	6.13	116.0471	0.76	3.93E+06	4.20E+05	
203.1103	87.0632	6.19	116.0471	1.22	2.45E+05	1.03E+04	
205.0898	89.0424	2.94	116.0473	0.24	2.65E+05	9.47E+03	
221.0503	105.0032	2.78	116.0471	0.71	1.02E+06	6.70E+04	
228.0975	112.0503	3.12	116.0472	0.54	2.31E+07	6.30E+06	2'-Deoxycytidine, dC[c]
229.0814	113.0345	4.74	116.0470	1.43	1.05E+07	9.13E+05	2'-Deoxyuridine, dU
229.1003	113.0534	3.12	116.0468	1.97	1.68E+06	2.69E+05	
230.0846	114.0379	4.74	116.0467	2.45	7.69E+05	1.74E+04	
237.0974	121.0505	5.16	116.0469	1.64	1.69E+05	2.03E+05	
239.1132	123.0662	5.19	116.0470	1.09	7.59E+05	5.41E+05	no ID
241.1294	125.0820	5.19	116.0474	0.35	3.60E+05	1.96E+05	
242.1129	126.0661	3.40	116.0468	1.88	5.91E+05	1.83E+05	5-Methyl-2'-deoxycytidine, 5-Me-dC
243.0968	127.0501	6.96	116.0468	2.25	7.55E+07	7.98E+06	Thymidine, dT[c]
244.0944	128.0470	6.96	116.0474	0.21	3.60E+05	4.51E+04	5-Hydroxy-2'-deoxycytidine, 5-OH-dC
244.0999	128.0535	6.96	116.0464	3.48	7.59E+06	4.16E+05	
247.1147	131.0683	6.13	116.0464	3.55	4.33E+04	2.69E+04	
247.1283	131.0813	5.67	116.0470	1.09	7.96E+06	3.70E+04	
248.1232	132.0766	2.86	116.0466	2.79	4.43E+06	5.96E+05	
251.0637	135.0163	4.71	116.0474	0.28	2.45E+07	2.21E+06	
252.0666	136.0200	4.71	116.0466	2.61	1.96E+06	8.59E+04	
252.1081	136.0613	5.16	116.0468	2.00	1.57E+09	9.60E+08	2'-Deoxyadenosine, dA[c]
253.0924	137.0455	6.30	116.0469	1.58	5.11E+06	4.56E+05	2'-Deoxyinosine, dI
253.1271	137.0787	9.94	116.0484	4.31	2.47E+05	2.86E+05	
254.0961	138.0488	6.30	116.0473	0.07	4.02E+05	1.94E+04	
254.1147	138.0684	5.16	116.0463	3.76	3.44E+06	9.94E+05	
254.1233	138.0771	5.16	116.0462	4.50	1.35E+07	7.80E+06	
255.1080	139.0613	6.30	116.0467	2.30	1.58E+05	1.17E+05	
256.1392	140.0926	5.19	116.0466	2.87	1.88E+06	2.98E+05	
256.1651	140.1181	3.48	116.0470	1.15	1.24E+06	8.64E+04	
257.1125	141.0658	6.93	116.0467	2.35	9.17E+05	6.49E+04	
260.1238	144.0762	3.09	116.0475	0.88	1.65E+06	4.70E+05	
261.1304	145.0836	9.91	116.0468	1.90	1.08E+07	1.65E+05	
265.0791	149.0320	6.96	116.0471	0.92	1.22E+08	7.82E+06	Na adduct of dT[c]
266.0764	150.0288	6.93	116.0475	0.91	6.61E+05	4.11E+04	
266.0822	150.0352	6.96	116.0470	1.13	1.27E+07	3.86E+05	
266.1239	150.0771	8.22	116.0468	1.92	2.97E+05	2.17E+05	N^6-Methyl-2'-deoxyadenosine, N^6-Me-dA
268.1034	152.0562	6.49	116.0473	0.04	2.82E+08	8.35E+07	2'-Deoxyguanosine, dG[c]
269.1007	153.0537	6.49	116.0470	0.93	3.46E+06	1.36E+06	
269.1066	153.0596	6.49	116.0470	1.20	2.94E+07	3.97E+06	
270.1078	154.0607	6.49	116.0471	0.75	1.17E+06	8.88E+04	
270.1182	154.0717	4.36	116.0465	2.81	1.55E+07	6.49E+04	
270.1189	154.0719	6.49	116.0469	1.41	2.35E+06	9.31E+05	
271.0892	155.0424	2.78	116.0467	2.06	5.64E+05	1.71E+05	
271.1222	155.0752	6.46	116.0470	1.22	1.37E+05	6.40E+04	
271.1391	155.0923	5.16	116.0468	1.78	7.05E+05	2.70E+05	
272.2204	156.1742	13.48	116.0462	4.22	3.56E+05	3.70E+03	
273.1071	157.0608	6.91	116.0463	3.54	8.74E+04	7.86E+03	

274.0904	158.0433	5.16	116.0471	0.85	9.74E+06	8.80E+04	Na adduct of dA[c]
274.1134	158.0668	6.54	116.0466	2.51	3.22E+05	1.39E+05	Guanidinohydantoin, Gh
275.0746	159.0275	6.33	116.0471	0.73	1.01E+06	5.86E+05	Na adduct of dI[c]
275.1230	159.0760	6.93	116.0470	1.14	1.23E+06	3.35E+05	
277.0409	160.9930	2.76	116.0479	2.10	1.31E+06	9.96E+04	
281.1486	165.1018	12.71	116.0468	1.93	2.32E+06	5.85E+04	
282.1189	166.0719	6.49	116.0470	1.04	9.25E+05	6.96E+05	
282.1192	166.0718	8.46	116.0474	0.39	2.79E+06	1.37E+06	
283.1223	167.0753	8.49	116.0470	1.20	2.05E+05	8.73E+04	
283.1269	167.0808	3.09	116.0460	4.52	5.26E+05	1.51E+05	
284.0984	168.0512	7.95	116.0472	0.33	2.34E+05	1.29E+05	8-Oxo-7, 8-dihydro-2'-deoxyguanosine, 8-oxo-dG
284.1237	168.0762	6.99	116.0475	0.65	3.05E+05	1.31E+04	
284.1347	168.0874	5.19	116.0472	0.33	1.74E+07	6.89E+06	no ID
285.1312	169.0848	5.14	116.0464	3.29	1.48E+05	9.62E+04	
285.1380	169.0909	5.19	116.0471	0.78	1.86E+06	3.89E+05	
286.1502	170.1035	5.16	116.0466	2.33	3.34E+06	5.89E+05	
288.1293	172.0827	6.52	116.0466	2.39	5.27E+05	1.13E+05	
289.0394	172.9921	3.84	116.0473	0.07	8.53E+04	2.26E+04	
290.0856	174.0383	6.49	116.0473	0.04	1.73E+07	4.94E+06	Na adduct of dG[c]
290.1431	174.0950	5.01	116.0481	2.86	3.62E+05	5.47E+04	
290.1776	174.1313	3.09	116.0463	3.34	1.04E+07	1.01E+05	
291.0821	175.0354	6.49	116.0468	1.85	3.99E+04	1.23E+05	
291.0828	175.0354	6.46	116.0474	0.40	8.33E+04	1.23E+05	
291.0888	175.0416	6.49	116.0472	0.30	1.49E+06	3.77E+05	
291.1180	175.0715	6.93	116.0466	2.50	1.54E+05	3.89E+03	
292.1129	176.0660	2.89	116.0469	1.37	2.25E+06	7.99E+05	
292.1625	176.1156	3.12	116.0469	1.28	8.21E+05	1.24E+05	
293.1167	177.0690	2.89	116.0477	1.34	1.68E+05	4.33E+04	
298.1138	182.0672	5.19	116.0466	2.28	2.04E+06	5.11E+05	
299.1094	183.0629	12.68	116.0465	2.69	1.61E+05	1.14E+04	
299.1343	183.0876	5.19	116.0466	2.27	4.56E+05	1.91E+05	
300.1295	184.0825	5.16	116.0470	1.01	2.13E+07	7.64E+06	no ID
300.1296	184.0825	6.46	116.0471	0.72	1.46E+06	2.74E+05	no ID
301.1135	185.0666	6.30	116.0469	1.28	3.54E+05	6.08E+04	
301.1327	185.0861	5.16	116.0467	2.12	1.85E+06	4.17E+05	
302.1451	186.0980	6.49	116.0470	0.84	6.07E+05	2.02E+05	
302.1454	186.0981	5.19	116.0473	0.07	3.95E+05	6.59E+04	
304.1011	188.0538	8.46	116.0473	0.05	9.35E+04	9.16E+04	
306.0595	190.0121	6.46	116.0474	0.45	7.37E+05	5.04E+04	no ID
306.1289	190.0822	2.89	116.0467	1.90	6.77E+06	1.32E+04	
307.1494	191.1023	3.66	116.0471	0.57	6.95E+05	4.60E+05	
308.1345	192.0874	3.92	116.0470	0.92	9.30E+04	8.95E+04	
309.1304	193.0832	7.40	116.0472	0.21	3.65E+04	1.75E+04	
310.1497	194.1032	2.83	116.0465	2.60	1.10E+06	8.80E+04	
311.0718	195.0249	6.99	116.0469	1.34	1.69E+06	1.79E+04	
313.0339	196.9862	4.74	116.0476	1.10	2.22E+06	4.57E+04	
314.1084	198.0616	6.49	116.0468	1.72	7.55E+05	9.96E+04	
314.1087	198.0617	5.37	116.0470	0.98	1.32E+05	5.09E+04	
314.2308	198.1848	13.59	116.0459	4.37	1.90E+05	6.86E+04	
315.0265	198.9797	6.96	116.0468	1.52	2.54E+05	1.62E+04	
315.1288	199.0822	5.21	116.0465	2.41	1.56E+05	7.63E+04	
316.1244	200.0776	6.52	116.0468	1.57	3.15E+06	4.74E+05	
316.1605	200.1139	5.19	116.0467	1.98	9.86E+05	1.47E+05	
317.1278	201.0809	6.52	116.0469	1.26	3.31E+05	2.35E+04	
318.9775	202.9307	4.74	116.0468	1.71	6.00E+05	8.72E+03	
320.1182	204.0713	2.78	116.0468	1.44	1.82E+05	1.71E+04	
321.1840	205.1373	6.30	116.0468	1.69	7.95E+04	3.49E+03	
324.1293	208.0826	8.89	116.0467	1.84	1.06E+07	6.17E+06	
327.0491	211.0020	6.96	116.0471	0.54	1.04E+07	1.77E+05	no ID
328.1606	212.1137	2.86	116.0469	1.20	1.79E+05	3.05E+04	
329.0059	212.9585	4.71	116.0473	0.12	1.58E+06	1.68E+04	
330.2071	214.1600	9.58	116.0471	0.54	1.70E+06	4.26E+03	
331.0425	214.9953	6.93	116.0473	0.10	1.45E+05	5.17E+04	
331.0439	214.9971	4.74	116.0468	1.51	1.79E+05	1.06E+05	
332.1195	216.0722	4.36	116.0473	0.14	1.52E+05	1.12E+04	
332.1550	216.1089	4.36	116.0461	3.61	1.09E+06	1.46E+04	

332.1552	216.1088	5.21	116.0464	2.60	1.74E+05	4.68E+04	
332.9925	216.9460	6.99	116.0464	2.58	1.92E+06	5.08E+04	
333.1837	217.1367	6.52	116.0470	0.95	3.05E+07	2.71E+05	
333.2015	217.1546	12.53	116.0469	1.15	3.16E+05	1.96E+05	
334.9893	218.9429	6.93	116.0464	2.60	4.43E+05	6.58E+03	
335.1634	219.1167	5.54	116.0467	1.83	9.02E+05	8.63E+04	
336.1838	220.1357	10.82	116.0481	2.46	1.97E+05	5.25E+04	
338.1333	222.0867	9.39	116.0465	2.24	9.04E+05	6.57E+04	
339.1207	223.0745	4.07	116.0462	3.22	1.06E+05	8.02E+04	
341.0650	225.0168	6.96	116.0481	2.46	5.62E+04	5.53E+03	
342.0045	225.9573	5.16	116.0472	0.24	1.04E+05	3.13E+04	
342.0136	225.9666	6.96	116.0471	0.72	6.30E+06	6.61E+04	
343.0219	226.9747	6.99	116.0472	0.42	5.17E+06	5.09E+04	
343.1598	227.1134	3.09	116.0464	2.73	3.40E+06	1.22E+06	
343.2047	227.1583	12.50	116.0465	2.36	1.35E+06	3.17E+05	
345.0600	229.0132	6.93	116.0468	1.42	6.60E+05	1.85E+05	
346.1344	230.0886	6.49	116.0458	4.34	6.87E+05	3.36E+04	
346.2077	230.1606	2.83	116.0471	0.61	1.53E+06	1.66E+04	
346.2079	230.1607	2.20	116.0472	0.18	6.39E+05	9.83E+03	
348.1396	232.0923	3.04	116.0473	0.03	4.01E+07	4.41E+04	
350.1710	234.1232	8.78	116.0478	1.57	2.84E+05	1.99E+05	
351.9923	235.9451	6.99	116.0472	0.36	7.28E+04	1.67E+04	
352.0332	235.9862	5.19	116.0470	0.72	1.70E+05	2.30E+04	
352.0554	236.0085	6.49	116.0469	1.18	8.16E+05	3.50E+05	
353.1715	237.1230	3.76	116.0485	3.33	3.05E+05	5.23E+04	
356.0289	239.9820	6.96	116.0469	1.03	8.27E+05	1.46E+04	
357.0740	241.0269	2.78	116.0471	0.65	4.01E+05	6.16E+04	
357.9984	241.9518	6.49	116.0466	1.95	1.74E+05	1.08E+05	
362.1920	246.1443	4.71	116.0476	0.88	1.25E+07	2.94E+05	
363.1948	247.1476	4.69	116.0472	0.27	1.55E+06	2.89E+04	
364.1341	248.0868	3.25	116.0473	0.11	3.24E+05	1.63E+05	
364.9953	248.9492	4.69	116.0461	3.19	2.55E+05	7.35E+03	
365.2174	249.1707	4.77	116.0467	1.59	1.80E+06	6.04E+03	
366.1660	250.1194	9.72	116.0466	1.98	2.31E+07	8.18E+03	
367.0197	250.9731	4.36	116.0466	1.94	7.57E+05	8.68E+03	
367.1598	251.1129	3.66	116.0469	1.05	8.79E+05	6.35E+05	
368.0280	251.9813	6.46	116.0468	1.47	3.36E+05	6.57E+04	
368.1195	252.0727	10.37	116.0468	1.29	8.69E+05	4.30E+05	
368.1546	252.1081	3.12	116.0465	2.16	4.98E+05	1.65E+05	
368.1651	252.1165	3.66	116.0486	3.42	8.55E+04	5.98E+04	
369.1661	253.1179	12.22	116.0482	2.54	1.64E+06	2.37E+05	

Table S2. Putative DNA adducts found in *M. affinis* using a manual approach (Gorokhova *et al.*[6]) that were not detected in the samples analyzed in this work.

**Precursor *m/z***	**Specific fragment *m/z***	**Retention time (min)**	**Adducts detected in earlier work (Gorokhova *et al.*[16]) and their proposed identification (ID)**
236.1281	120.0809	8.48	no ID
252.1228	136.0759	11.3	no ID
268.1043	152.0569	6.41	8-Hydroxy-2′-deoxyadenosine, 8-OH-dA
278.1605	162.1126	3.59	no ID
289.1758	173.1288	5.60	no ID
295.1203	179.0739	3.51	no ID
